# Supplementary material for: A Cationic Amphiphilic AIE Polymer for Mitochondrial Targeting and Imaging
Source: Pharmaceutics. 2022 Dec 28;15(1):103. doi: 10.3390/pharmaceutics15010103 (PMC9866158; doi:10.3390/pharmaceutics15010103)
Supplement: Supplementary file 1 [file pharmaceutics-15-00103-s001.zip › pharmaceutics-1972873-supplementary.pdf]

Supporting Information

A Cationic Amphiphilic AIE Polymer for Mitochondrial Targeting and Imaging

Junliang Zhou, Haiyang Wang, Wen Wang, Zhiwei Ma, Zhenguo Chi and Siwei Liu *

PCFM Lab, GD HPPC Lab, School of Chemistry, Sun Yat-sen University, Guangzhou, Guangdong 510275, China

* Correspondence: liusiw@mail.sysu.edu.cn

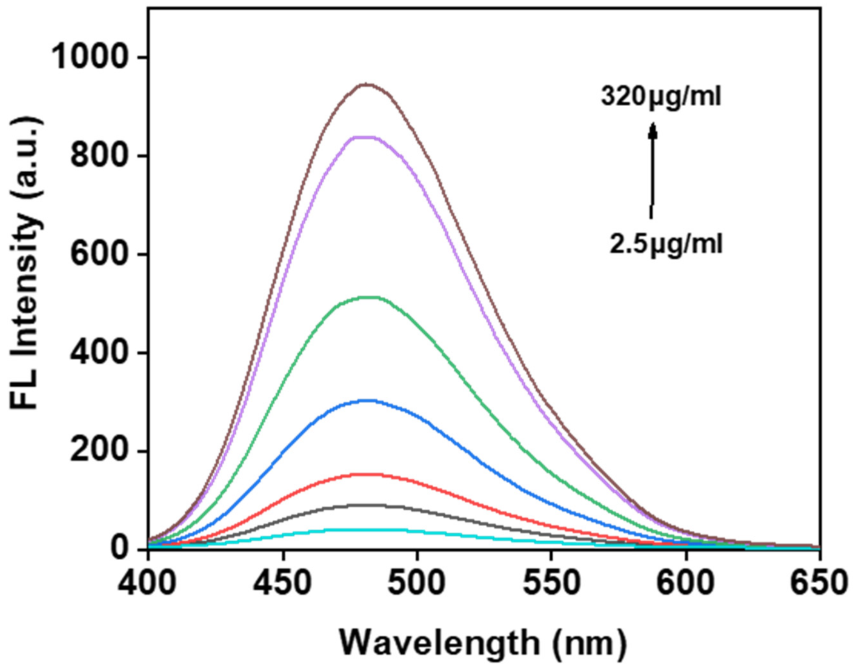

Figure S1. Fluorescence spectra of PTD with different concentrationsin PBS buffer: 2.5,5, 10, 20, 40, 80, 320μg/mL

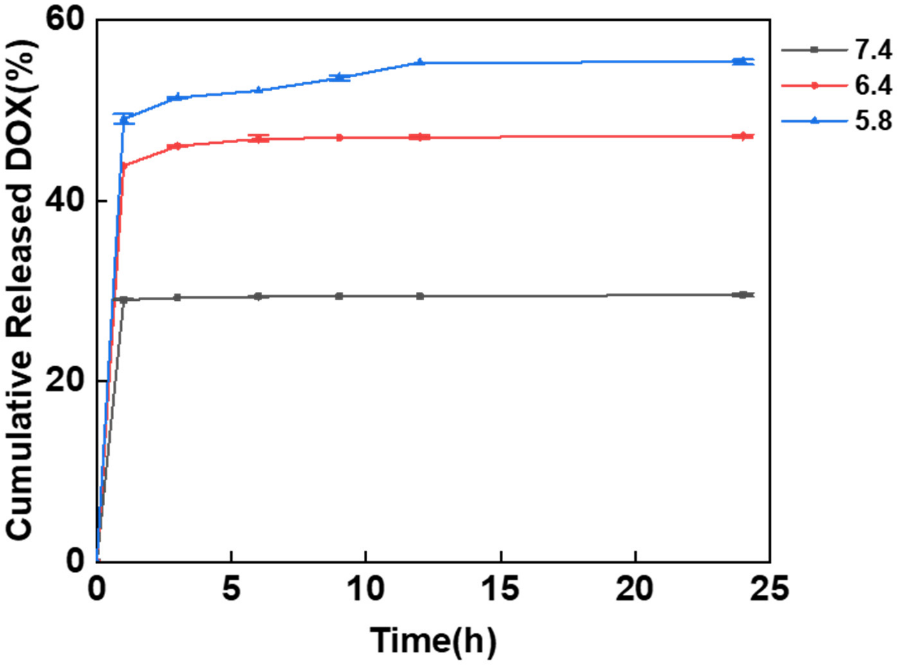

Figure S2. Cumilative release profile of DOX at different pH value(37°C)

According to the drug release curve, different pH environments can regulate the release of loaded DOX. It shows the tendency of first violent release and then slow release. The lower the pH surrounding the carrier, the faster the DOX release rate from the carrier.

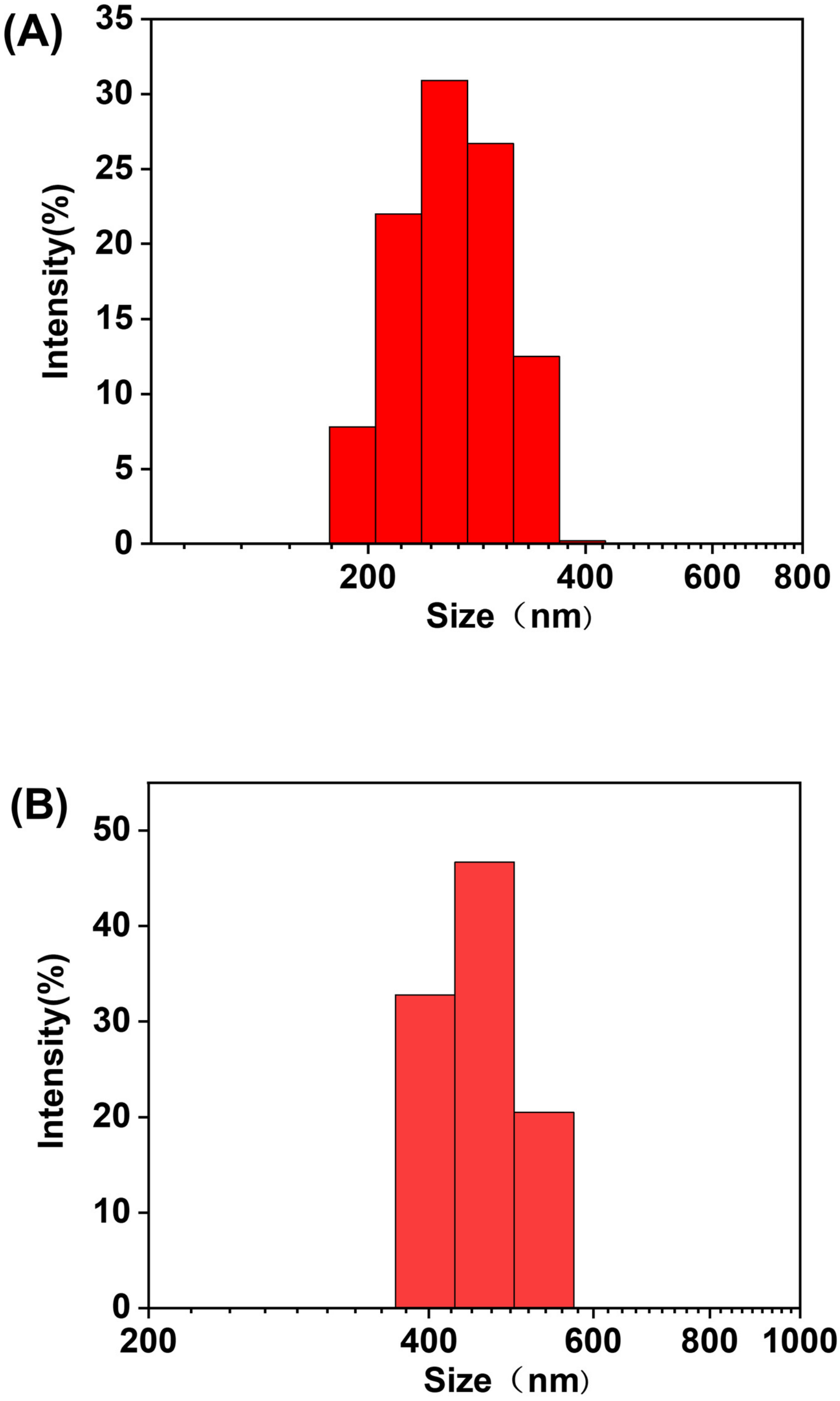

Figure S3. Particle size distribution histograms of (A) PTD and (B) DOX-loaded PTD
